# **Physiological regulation of oral saliva ion composition and flow rate are not coupled in healthy humans**—**Partial revision of our current knowledge required**

**DOI:** 10.1007/s00424-024-03025-9

**Published:** 2024-10-02

**Authors:** Gerald Schwerdt, Marie-Christin Schulz, Michael Kopf, Sigrid Mildenberger, Sarah Reime, Michael Gekle

**Affiliations:** Julius-Bernstein-Institute of Physiology, Martin-Luther-Universität Halle-Wittenberg, Magdeburger Straße 6, 06112 Halle (Saale), Germany

**Keywords:** Oral saliva, Physiological ion composition, Osmolality, PH, Physiological stimulation

## Abstract

Appropriate composition of oral saliva is essential for a healthy milieu that protects mucosa and teeth. Only few studies, with small sample numbers, investigated physiological saliva ion composition in humans. We determined saliva ion composition in a sufficiently large cohort of healthy adults and analyzed the effect of physiological stimulation. We collected saliva from 102 adults under non-stimulated and physiologically stimulated conditions (chewing). Individual flow rates, pH, osmolality, Na^+^, K^+^, Cl^−^, and HCO_3_^−^ concentrations under both conditions as well as the individual changes due to stimulation (Δvalues) were determined. Non-stimulated saliva was hypoosmolal and acidic. Na^+^, Cl^−^, and HCO_3_^−^ concentrations remained well below physiological plasma values, whereas K^+^ concentrations exceeded plasma values more than twofold. Stimulation resulted in a doubling of flow rates and substantial increases in pH, HCO_3_^−^, and Na^+^ concentrations. Overall, stimulation did not considerably affect osmolality nor K^+^ or Cl^−^ concentrations of saliva. An in-depth analysis of stimulation effects, using individual Δvalues, showed no correlation of Δflow rate with Δion concentrations, indicating independent regulation of acinar volume and ductal ion transport. Stimulation-induced Δ[Na^+^] correlated with Δ[HCO_3_^−^] and Δ[Cl^−^] but not with Δ[K^+^], indicating common regulation of ductal Na^+^, Cl^−^, and HCO_3_^−^ transport. We present a robust data set of human oral saliva ion composition in healthy adults and functional insights into physiological stimulation. Our data show (i) that flow-dependence exists for Na^+^ and HCO_3_^−^ but not for K^+^ and Cl^−^ concentrations, (ii) osmolality is flow-independent, (iii) regulation of Na^+^, Cl^−^, and HCO_3_^−^ transport is coupled, (iv) regulation of flow rate and ion concentrations are independent and (v) spatially separated between acini and ducts, respectively.

## Introduction

Oral saliva plays an important role in the maintenance of oral milieu homeostasis and functionality. Saliva facilitates food transportability during swallowing and allows the initial digestion of food. Saliva is also important for immune defense, degustation, and speech. By balancing pH, it protects the soft oral tissue and teeth from an acidic and cariogenic environment created by organic anions such as lactate, propionate, or formate [5]. The therein-contained growth factors are essential for the regeneration of oral mucosa [4]. Dysregulation of oral saliva production is of clinical relevance because it can affect esophageal pH-clearance, disturb oral mucosa integrity as well as dental mineral homeostasis, and lead to oral dysbiosis.

Under non-stimulated conditions, saliva is produced in a circadian rhythm [9] at low levels by acinar and duct cells of the major salivary glands as parotid (alpha-amylase-rich saliva), sublingual (mucous-rich) and submandibular (mucous and serous) glands [18]. Furthermore, minor salivary glands contribute to the final oral saliva composition [1, 11]. Upon several stimuli, like smell, taste, or chewing of food, the saliva production rate increases, controlled by the autonomous nervous system with muscarinic and beta-adrenergic receptors as intermediate signal transducers [20, 23]. The contribution of the different glands changes with stimulation, whereby the contribution of parotid glands increases [19].

Oral salivary glands are compound glands consisting of terminal parts (acini) and excretory ducts. Because the current knowledge concerning their function has been described and recently reviewed [7, 15, 18, 24], we will give only a brief introduction and refer to the literature cited in the reviews. The smallest functional secretory unit consists of an acinus and the associated secretory duct and produces a protein-rich, plasma-like fluid. The majority of the salivary proteins are synthesized in the acinar cells that produce the primary saliva with an electrolyte composition similar to plasma [25]. In the acini, anions (mainly Cl^−^ via apical ANO1 and basolateral NKCC1 and NHE1) are secreted actively, and Na^+^ (paracellular) and water (a.o. via AQP5) follow (together with a.o. K^+^ and HCO_3_^−^). This fluid is isotonic and has a similar electrolyte composition to plasma [6]. The extent of primary saliva formation is determined by the activity of apical Cl^−^ and basolateral K^+^ channels, which are also responsible for secretion regulation. Stimulators of saliva secretion lead to an increase in cytosolic Ca^2+^ concentration, which activates protein kinases that stimulate channel activity and fluid secretion. The regulation of acinar function occurs mainly through the autonomic nervous system. Acetylcholine and substance P trigger cytosolic Ca^2+^ oscillations via phospholipase C, which increases protein exocytosis and fluid secretion. ATP increases the cytosolic Ca^2+^ concentration by activating a cation channel (P_2Z_ purinoreceptor).

Primary saliva is modified in the excretory ducts to form secondary saliva. The duct cells of the salivary glands are polarized epithelial cells that transport electrolytes [7, 24]. The cells show a certain heterogeneity along the gland ducts. At the transition from the acinus to the secretory duct, cells with high carbonic anhydrase activity are found, which probably plays a role in HCO_3_^−^ secretion. Along the excretory ducts, Na^+^ is reabsorbed, and K^+^ and HCO_3_^−^ are secreted. Apical Na^+^ channels (ENaC) and Na^+^/H^+^ exchangers (NHE-2/-3) are involved in Na^+^ reabsorption. Apical Cl^−^/HCO_3_^−^ exchangers (e.g., Slc26a6) and Cl^−^ channels (e.g., CFTR) and basolateral Cl^−^ channels are involved in Cl^−^ transport. Cl^−^/HCO_3_^−^ exchange ensures HCO_3_^−^ secretion. HCO_3_^−^ derives from CO_2_ and is possibly taken up by basolateral Na^+^-cotransport (NBCe1B). The apical step of K^+^ secretion probably takes place via K^+^ channels. Since the ducts are relatively watertight, the osmolality of the secondary saliva becomes hypotonic. Because electrolyte transport in the duct epithelium is limited, changes in the ion composition of final saliva can also depend on changes in the acinar secretion rate.

In the non-stimulated state, secondary saliva is hypotonic, low in NaCl, and rich in K^+^. When the gland is stimulated, ion concentrations approach that of plasma, without ever reaching the same values, with the exception of HCO_3_^−^ that can reach concentrations above those in plasma, resulting in pH values up to 8. The most important regulator of the acini is the parasympathetic nervous system. Acetylcholine stimulates secretion via M_3_ receptors. Acini are also subject to control by the sympathetic nervous system (β_2_ receptors). Noradrenaline increases saliva flow and shifts its composition towards a less watery, viscous saliva (through disproportionately increased protein exocytosis). Some nerve fibers release substances P and VIP, which also stimulate saliva secretion. Regulation of excretory duct function is less well studied than that of the acinar cells. Acetylcholine leads to a decrease in NaCl reabsorption and K^+^ secretion but an increase in HCO_3_^−^ secretion. β_2_ receptors stimulate NaCl reabsorption. Aldosterone stimulates Na^+^ reabsorption and K^+^ secretion. This differential regulation of acini and excretory ducts opens the possibility of decoupled changes in saliva flow rate and saliva composition.

According to the hitherto view, the extent of net reabsorption in the excretory ducts is dependent on saliva flux rates. Sodium, chloride, or bicarbonate, for example, are reabsorbed less after flux stimulation, whereas potassium secretion is supposed to be enhanced at higher flux rates, leading in summary to increased saliva osmolality [18]. Furthermore, the secretion of different glands is stimulated by different stimuli [20] and they produce saliva of different composition concerning protein, mucin, or ion concentrations [8, 10]. Yet, crucial for an appropriate oral milieu is the total oral saliva composition which is a mixture due to the different portions of the different glands.

One of the original findings, which describes the alteration of ion composition due to flux rates and is the basis of our current understanding of saliva composition, dates back to a study by Thaysen et al. performed in 1954 [24]. This study presents data on flux-dependent ion composition of saliva that are the basis of our knowledge regarding prototypical total oral saliva composition until today. A later study by Bardow et al. presents similar results concerning the change of sodium, chloride, and bicarbonate concentrations upon stimulation of salivary flow [3]. Both describe a flow-dependent increase of parotid sodium, chloride, and bicarbonate concentrations and an almost unchanged potassium concentration. Bardow et al. additionally describe a flow-dependent increase of pH and of bicarbonate concentration.

Although the results of these initial studies are the basis of our current understanding of oral saliva composition, it must be kept in mind that these studies were performed with a limited number of participants and flux was stimulated by subcutaneous injection of beta-methyl-acetylcholine-hydrochloride [24] or by citric acid [3]. Thaysen et al. [24] used only three young women with uncomplicated essential hypertension for their study and Bardow et al. five healthy young males [3]. Thaysen et al. determined the composition of unstimulated and stimulated saliva from only one salivary gland (parotid), and therefore, the composition of the saliva in the whole oral cavity may differ due to the admixture of the other salivary glands, which is indicated by the findings of Bardow et al. [3].

In the present study, we present data regarding flux-dependent oral cavity saliva composition from 102 healthy young volunteers and compare them to the data recorded in older studies which are often the source of values also in textbooks. We used total oral cavity saliva to determine changes in osmolality, sodium, chloride, and bicarbonate concentration as well as pH before and after 5 min chewing a tasteless paraffin cube. Our results, at least in part, do not support the current understanding of saliva ion composition based on older studies, and therefore, the knowledge of ion composition of total oral cavity saliva should be revised.

## Materials and methods

### Sample recovery

Saliva was collected between 2 and 3 p.m. 102 healthy volunteers, (out of 300 3rd semester students during the course of physiology; age 19–31; 27% males, 73% females; only healthy students are allowed to serve as volunteers) were asked to collect the spontaneous saliva over 5 min under resting conditions by spitting into a calibrated vessel. This was followed by a second 5-min collecting period with stimulated saliva production by chewing a tasteless paraffin cube (patient wax, Ormco Corp., Glendore, CA, USA). Saliva was used immediately after collection for simultaneous analysis with no freezing or storing. Major debris was removed, and care was taken to use only aliquots for analysis that were free of debris. Collected saliva represents the total oral cavity saliva, which is relevant for milieu homeostasis, and not the saliva of a specific salivary gland. Since saliva was collected anonymously, we are not able to compare male and female volunteers.

### Saliva osmolality

Saliva osmolality was determined using a freezing point depression osmometer (Vogel MedTec GmbH, Gießen, Germany). One hundred microliter of saliva was used. For comparisons sake, we determined randomly blood plasma osmolality in some participants. These values were all within the expected range (290 to 300 mosm/kg).

### Sodium and potassium concentrations

Saliva was diluted 1000-fold with deionized water (20 µl in 20 ml), and sodium and potassium concentrations were determined by a flame photometer (BWB Technologies, Newbury, UK).

### Chloride concentration

Chloride concentration in saliva was determined chemically. Saliva was diluted fivefold with deionized water, and 10 µl of dilution was added to 490 µl chloride reagent (0.48 mmol/l Hg(NO_3_)_2_, 0.98 mmol/l 2,4,6-Tris(2-pyridyl)-s-triazin, 2.67 mmol/l H_2_SO_4_, 20.2 mmol/l Na_2_SO_4_, 0.44 mmol/l FeSO_4_) in a cuvette. After 15 min of incubation at room temperature, absorption was measured at 620 nm in a photometer.

### Ph and bicarbonate in saliva

pH of saliva was determined using pH indicator stripes in the range from 6.0 to 8.1 (VWR Chemicals, Leuven, Belgium). Bicarbonate concentration was calculated using the Hendersen-Hasselbalch equation ([HCO_3_^−^] = [CO_2_] × 10^(pH−6.1)^ with 6.1 as the pKa at 37 °C). The concentration of CO_2_ was calculated using a PCO_2_ in unstimulated saliva of 36 mmHg (1.08 mmol/l) and in stimulated saliva of 32.16 mmHg (0.96 mmol/l) according to the findings of Bardow et al. and others [3, 14].

### Ethics approval statement

The Ethics Committee of the Martin Luther University Halle-Wittenberg (2023–185) approved the study.

### Statistic

Pre-study data obtained during the design of the study were used to calculate the required minimal number of participants. With a ratio (differences of means)/(standard deviation) around 1.7, power = 0.800, and alpha = 0.01 the minimum sample size was 70. Normality testing of the data was performed using the Shapiro–Wilk test. The results showed that the majority of parameters are not normally distributed. Therefore, we used a distribution-independent, non-parametric test procedure (Wilcoxon signed-rank test) to test between unstimulated and stimulated saliva. The pH values were also transformed to [H^+^]-values for additional statistical testing. For pairwise comparisons, the threshold for statistically significant differences was set to *p* < 0.05. All statistical calculations and regression analysis were performed with SigmaPlot 12.5 (Alfasoft GmbH, 60327 Frankfurt am Main, Germany) using linear regression to obtain the Pearson correlation coefficient.

## Results

### Saliva flow rate and osmolality

**Fig. 1:**
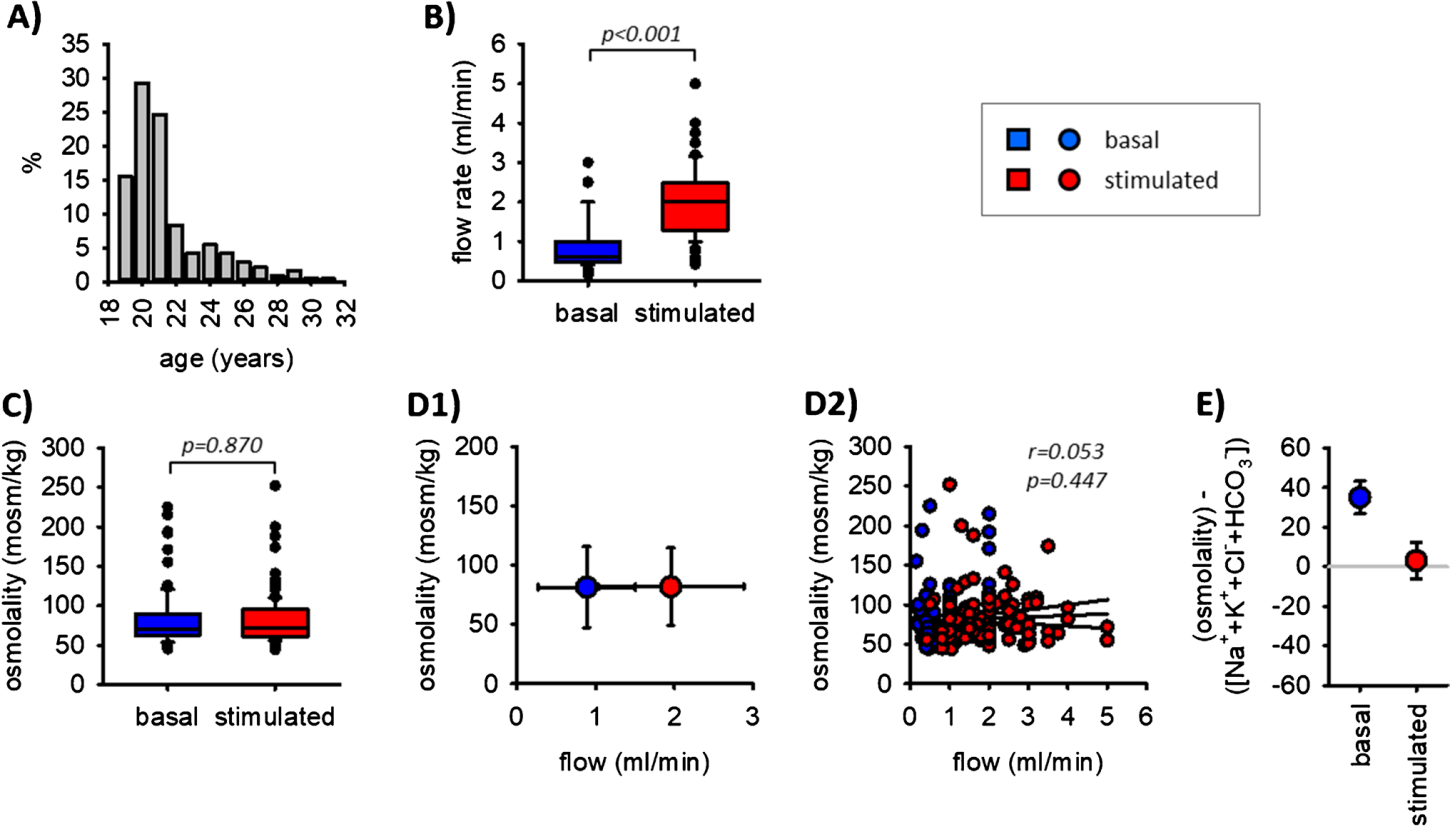
Age distribution of participants (**A**). Saliva flow rate (**B**), saliva osmolalities (**C**), and flow-dependent osmolalities (**D1** and **D2**) before and during chewing. *n* = 102. In (**E**) the gap between total osmolality and the sum of the osmolytes Na^+^, K^+^, C^l−^, and HCO_3_^−^ is shown for basal and stimulated saliva flow

As shown in Fig. 1B and Table 1, the saliva flow rate increased during chewing significantly from 0.9 to 2.0 ml/min. However, the osmolality of oral cavity saliva remained almost unaffected from chewing (82 mosm/kg before and 82 mosm/kg after chewing, Fig. 1C), did not correlate with flow rate, and was always clearly below blood plasma and primary saliva osmolality (291 ± 4 mosm/kg [6]). Thus and in contrast to previous studies, the oral cavity saliva osmolality was independent of the flow rate in the range of physiological stimulation.

**Table 1:** Salivary flow, osmolalities, ion concentrations, and pH found in our study (mean ± S.E.M.). Number of samples in brackets. The asterisk (*) indicates significant difference to unstimulated values. The number of samples for chloride and sodium is lower because some measurements had to be discarded for technical reasons as improperly allocated data, wrong dilutions, or transfer to the data file.

	Unstimulated	Median	Stimulated	Median
Flow (ml/min)	0.9 ± 0.1 (102)	0.6	2.0 ± 0.1 (102)*	2.0
Osmolality (mosm/kg)	82 ± 3 (102)	71	82 ± 3 (102)	73
Na^+^ (mmol/l)	3.6 ± 0.3 (100)	2.7	10.3 ± 0.8 (100)*	7.2
K^+^ (mmol/l)	13.7 ± 0.8 (102)	11.2	15.1 ± 0.6 (102)*	14.8
Cl^−^ (mmol/l)	21.2 ± 2.5 (86)	12.1	22.4 ± 2.6 (86)	14.5
pH	6.81 ± 0.03 (102)	6.9	7.45 ± 0.03 (102)*	7.5
Free H^+^ (nmol)	198 ± 16 (102)	126	47 ± 4 (102) *	32
Calculated HCO_3_^−^ (mmol/l)	7.2 ± 0.5 (102)	6.8	28.5 ± 2.3 (102)*	24.2

When we compared saliva osmolality with the sum of the osmolytes Na^+^, K^+^, Cl^−^, and bicarbonate, we obtained a gap (osmolality minus the sum of ions) of 33 ± 8 mosm/kg (median ± 95% confidence interval, Fig. 1E) under control conditions. By contrast, when saliva production was stimulated, this gap disappeared completely (8 ± 9 mosm/kg). Thus, under stimulated conditions, there is no discrepancy regarding osmolality and ion composition.

### Sodium and potassium in saliva

**Fig. 2:**
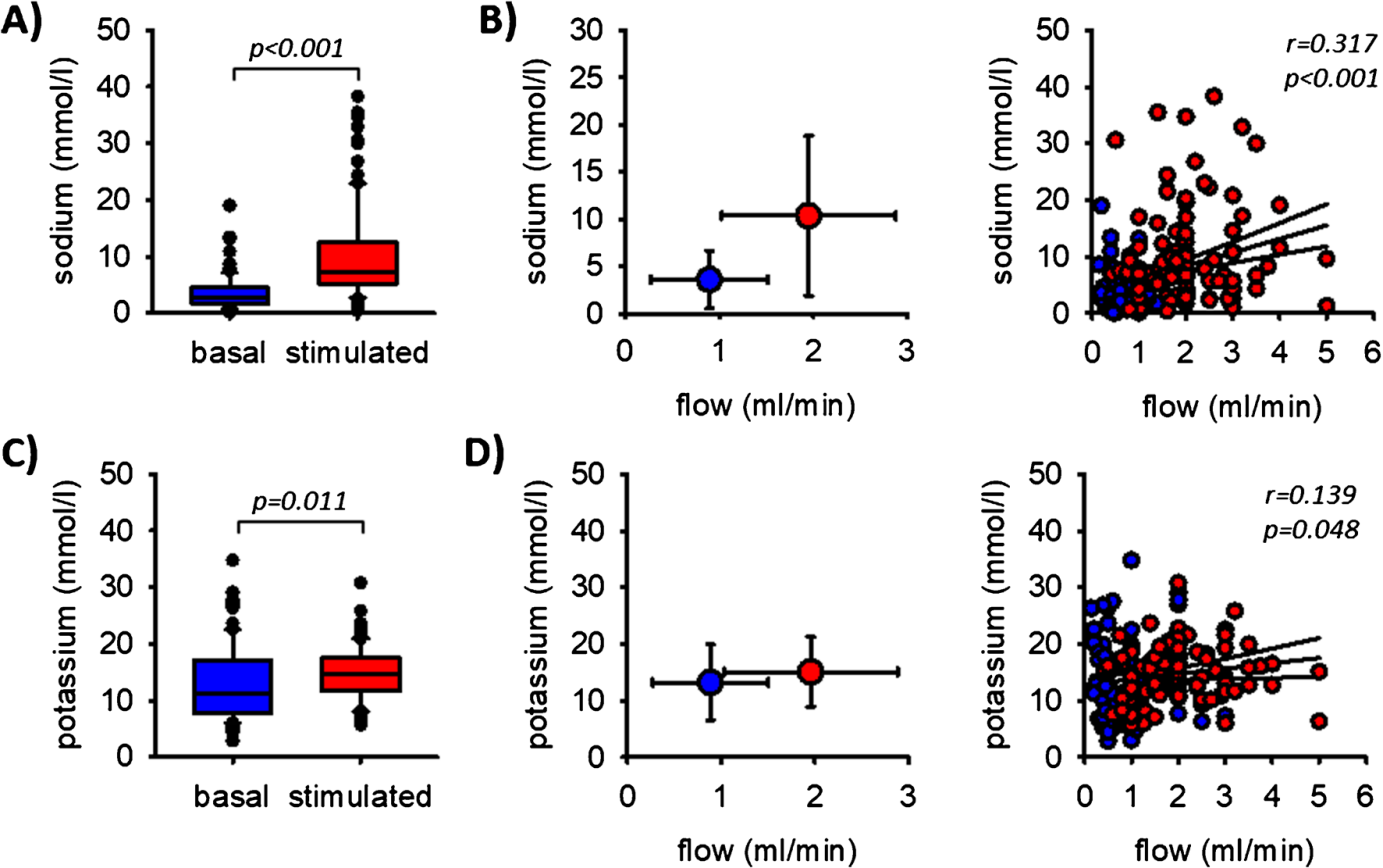
Sodium (**A**) and potassium (**C**) concentrations in saliva before and during chewing and flow-dependence (**B**, **D**). The lines indicate the linear regression (middle one) and the 95% confidence interval. *n* = 102 for potassium and *n* = 100 for sodium

In unstimulated oral cavity saliva, we determined a sodium concentration of 3.6 mmol/l which increased significantly to 10.3 mmol/l by chewing, Fig. 2A). The higher the flow rate, the higher the concentration of sodium (i.e., there is a significant correlation, Fig. 2B) although the concentrations remained always well below physiological blood plasma sodium concentration, which is about 140 mmol/l. The values are also lower compared to the concentrations reported for parotid saliva by Thaysen et al. [24]. In sum, stimulation led to an approx. tenfold increase in sodium excretion rate (Table 2). The potassium concentration was almost not affected by chewing, whereat the small increase from 13.7 to 15.1 mmol/l was statistically significant. It did not correlate with the flow rate (Fig. 2C, D). Consequently, the salivary potassium excretion rate doubled during chewing (Table 2). In both, unstimulated and stimulated saliva, the potassium concentrations were in most cases well above physiological blood plasma concentrations.

**Table 2:** Salivary excretion rates determined in our study (mean ± S.E.M.). The numbers in brackets represent the number of samples. Secretion rate of substance X = saliva flow rate × concentration of substance X in this portion of saliva. The asterisk (*) indicates significant difference to unstimulated values. The number of samples for chloride and sodium is lower because some measurements had to be discarded for technical reasons as improperly allocated data, wrong dilutions, or transfer to the data file.

	Unstimulated	Median	Stimulated	Median
Na^+^ (µmol/min)	2.8 ± 0.3 (100)	2.0	20.8 ± 2.2 (100)*	13.3
K^+^ (µmol/min)	12.4 ± 1.3 (102)	7.6	30.7 ± 2.0 (102)*	27.7
Cl^−^ (µmol/min)	15.8 ± 2.3 (86)	6.9	38.8 ± 6.5 (86)*	17.8
HCO_3_^−^ (µmol/min)	6.4 ± 0.7 (102)	3.7	58.8 ± 6.0 (102)*	37.0

### Bicarbonate and protons

**Fig. 3:**
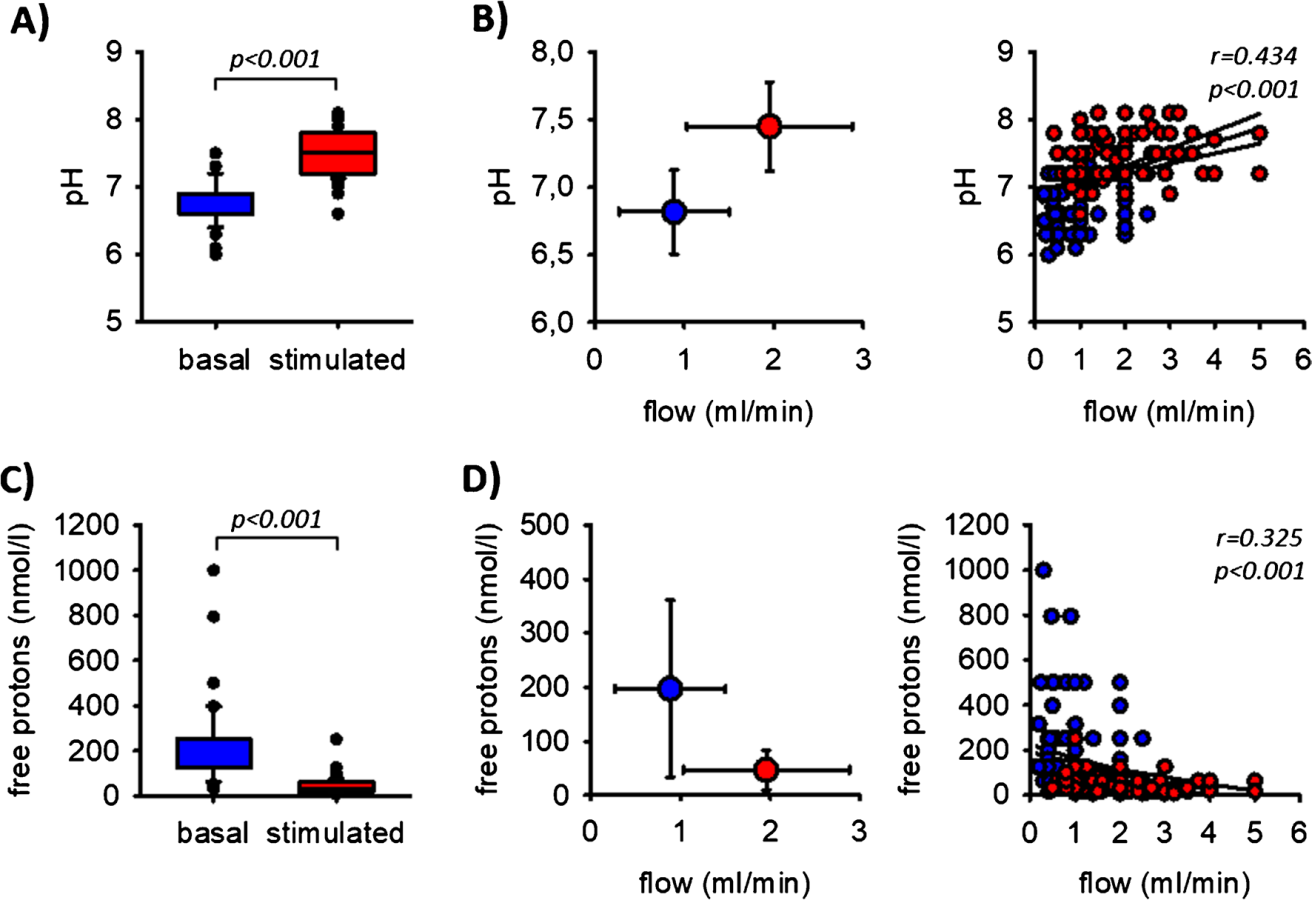
**A** Saliva pH before and during stimulation. **B** Flow-dependence of saliva pH. **C** Free proton concentrations in saliva before and during stimulation. **D** Flow-dependence of free proton concentrations. The lines indicate the linear regression (middle one) and the 95% confidence interval. *n* = 102

The pH value of saliva increased significantly from 6.81 to 7.45 when saliva production was stimulated (Fig. 3A). Accordingly, the calculated free proton concentration decreased significantly (Fig. 3C). The pH value and therefore the free proton concentration correlated significantly with the flow rate (Fig. 3B and D).

**Fig. 4:**
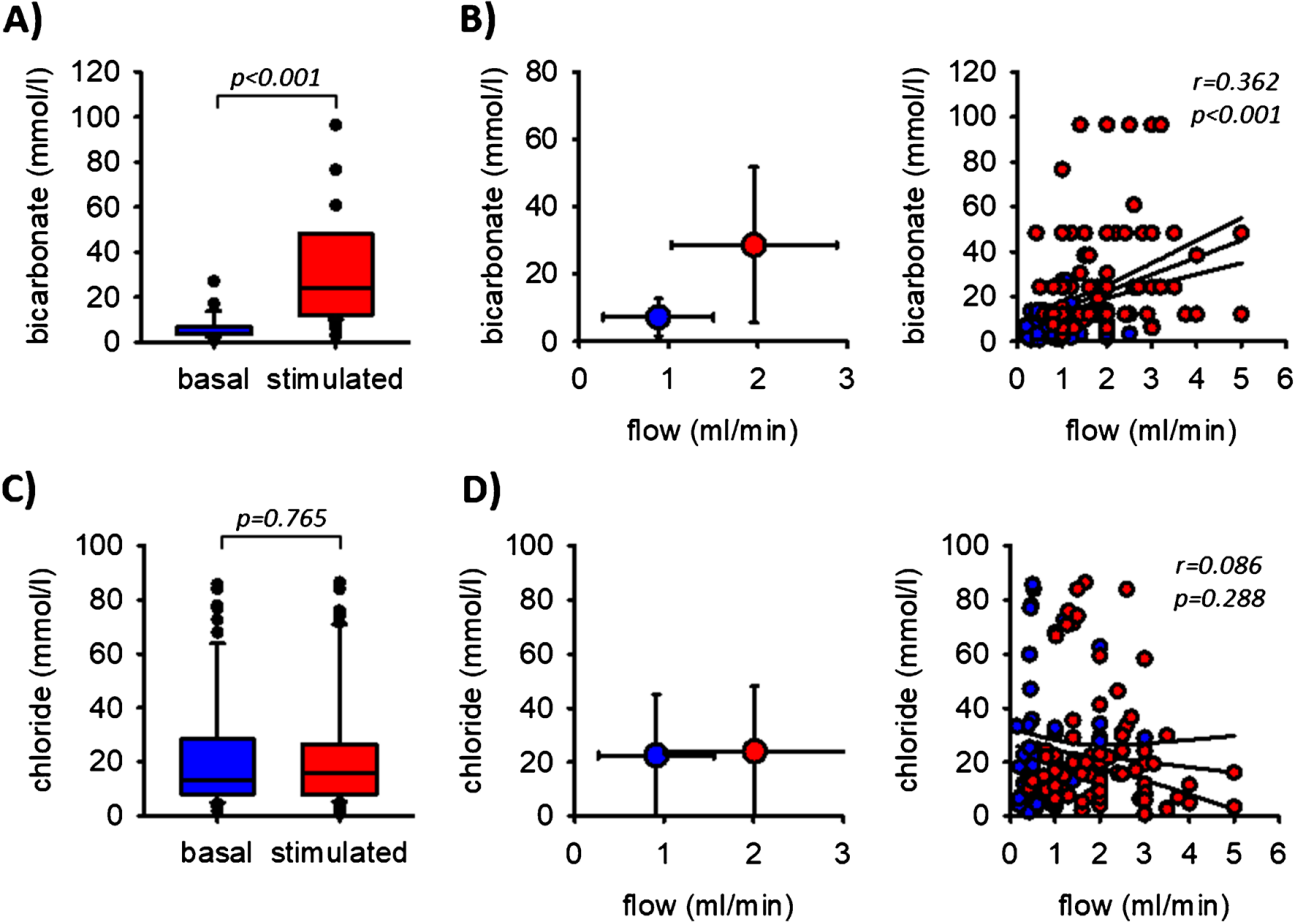
**A** Bicarbonate concentrations in saliva before and during stimulation. **B** Flow-dependence of bicarbonate concentrations. *n* = 102. **C** Chloride concentrations in saliva before and during stimulation. **D** Flow-dependence of chloride concentrations. The lines indicate the linear regression (middle one) and the 95% confidence interval. *n* = 86

The bicarbonate concentration increased from 7.2 to 28.5 mmol/l (Fig. 4A). Therefore, the bicarbonate concentration in oral cavity saliva can exceed blood plasma bicarbonate concentration when stimulated, indicating an active secretion mechanism along the duct. Because bicarbonate concentration correlated significantly with the flow rate (Figs. 4B), the bicarbonate excretion rate increased approx. ninefold during chewing (Table 2).

### Chloride in saliva

Chloride concentration was not affected significantly by chewing (21.2 mmol/l before and 22.4 mmol/l after chewing, Fig. 4C) and did not correlate with flow rate (Fig. 4D). Thus, the chloride concentration was independent of saliva flow in the physiological range and was always clearly below blood plasma chloride concentrations (120 mmol/l). In sum, the chloride excretion rate doubled during chewing (Table 2).

### Stimulation of flow rate and changes in ion composition are not coupled

**Fig. 5:**
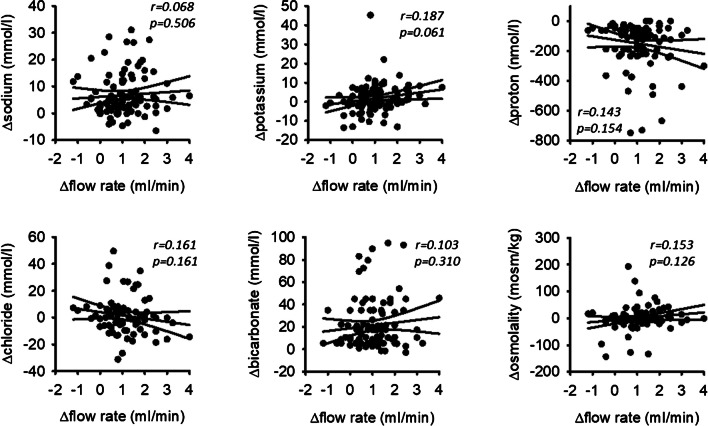
Chewing-induced changes in flow rate (Δflow rate) do not correlate with chewing-induced changes in ion composition (Δion concentration). *N* = 86 for chloride, *n* = 100 for sodium, *n* = 102 for all others. The lines indicate the linear regression (middle one) and the 95% confidence interval

Correlations of absolute flow rates with ion concentrations do not allow conclusions regarding a direct coupling of saliva flow stimulation (i.e., stimulation of the acini) with the stimulation of changes in ion composition that result from epithelial transport in the duct system. Therefore, we performed an in-depth analysis on the individual changes in flow and ion concentration elicited by chewing. Figure 5 shows the effects of stimulation on the individual changes in ion concentrations (i.e., individual Δ values) as a function of individual changes in flow rate (Δflow rate). There was no significant correlation between Δflow rate and Δion concentration or Δosmolality, indicating an independent regulation of flow rate in the acini and ion transport (especially sodium, bicarbonate, and protons) in the duct system. Thus, the changes in ion composition are not the result of altered flow rates.

### Stimulation of changes in sodium, bicarbonate, and chloride concentration are coupled

**Fig. 6:**
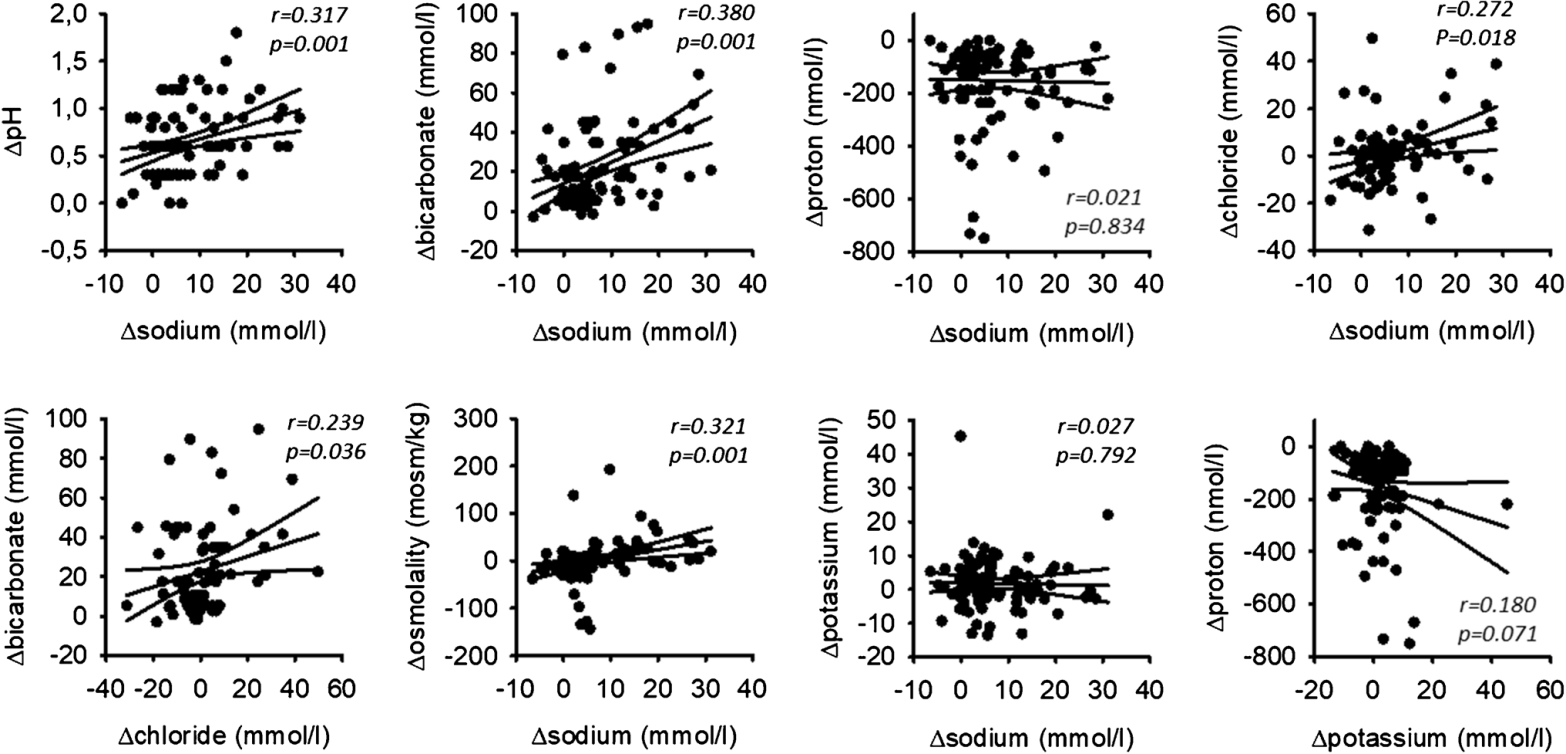
Correlation of chewing-induced changes in ion composition (Δion concentration). *N* = 86 for chloride, *n* = 100 for sodium, *n* = 102 for all others. The lines indicate the linear regression (middle one) and the 95% confidence interval

Finally, we investigated whether the stimulation-induced changes of individual ion concentrations are coupled. Figure 6 shows the correlation of the individual effects of stimulation on ion composition. The stimulation-induced increase in sodium concentration correlated significantly with the changes in bicarbonate and chloride concentration, as well as with osmolality, but not with the changes in potassium concentration. In addition, the changes in bicarbonate and chloride concentrations also correlated significantly. These data strongly suggest a functionally coupled transport of sodium, bicarbonate, and chloride in the salivary duct system, which is not coupled to potassium transport.

## Discussion

Oral saliva is an important fluid with a variety of functions, including partial or initial digestion of food, oral hygiene, and facilitation of swallowing or speaking. Various stimuli, such as chewing or smell of food or even imagination of a favorite meal (mouthwatering) lead to increased saliva production, i.e., saliva flow rate. According to literature, also the ion composition of saliva is affected when flow rates increase due to stimulation. Our knowledge on saliva composition and flow-dependent changes is mostly based on studies performed even decades ago with a limited number of human participants or even in animals such as rats [16, 17]. To gain insight into saliva composition from a larger healthy human cohort, we determined the ion composition of unstimulated and stimulated whole oral cavity saliva from 102 healthy volunteers. Participants were asked to induce salivary flow by chewing a tasteless paraffin cube for 5 min, and the composition of saliva was determined before and during flow stimulation. As the results had to be rendered totally anonymous, we were not allowed and not able to distinguish between male and female samples. Therefore, a possible sex-specific effect remains so far undetected as well as a conceivable influence of the menstrual cycle on saliva composition, as was shown in 20 Murrah buffaloes [12], remains open. Of course, these questions are interesting and of possible relevance. Therefore, separate studies have to be designed and conducted that allow further sub classifications. With these future studies, the questions regarding the influence of sex and menstrual cycle can be addressed.

Stimulation of saliva production by chewing increased flow rates by a factor of ~ 2. Surprisingly, and in contrast to previous studies, in our cohort with 102 volunteers osmolality did not change significantly during stimulation, did not correlate with flow rate, and remained with few exceptions well below 100 mosm/kg. A low initial or unstimulated osmolality was reported in a study performed with 65 children. Unfortunately, no flow-induced saliva was collected and analyzed in that study [21]. A study with rats showed an initial steep decrease of osmolality from 260 mosm/kg down to 100 mosm/kg followed by an increase up to 150 mosm/kg [17], cited by [22]. We also found that under stimulated conditions there is no discrepancy regarding osmolality and ion composition. The explanation for the osmolality gap under control conditions, i.e., when the saliva flow rate is low, is the presence of osmotic active substances that are not derived from saliva but, e.g., from microbiome metabolism or ingested food. Furthermore, oral saliva contains calcium, magnesium, phosphates urea, and ammonia [2]. These “non-primary salivary” compounds contribute significantly to osmolality only at low flow rates and are no longer interfering when flow rate increases, leading to their dilution. Overall, our finding that the human oral cavity saliva osmolality does not correlate with flow rate nor changes during physiological stimulations is in contrast to our hitherto understanding, which is mainly based on studies using rats that often reported a U-shaped flow-dependent osmolality [16, 17].

We observed similar differences between published data and ours for potassium and chloride concentrations. According to data in literature [24], potassium concentration should decrease from 30 to 15 mmol/l or remain constant with a tendency to lower concentrations at higher flow rates. In our study, using oral cavity saliva, we did not observe a decrease. Studies by Dawes from 1969 and 1974 show that the potassium concentration in parotid saliva decreases upon stimulation, whereas in submandibular saliva potassium concentrations remain almost unaltered [8, 10]. In the same studies, chloride concentration decreases upon stimulation in parotid saliva, but increases in submandibular saliva. The stimulation of both and the admixture of the minor glands [11] leads to almost unchanged chloride concentration in oral cavity saliva, as shown by our results. It is therefore necessary to know the origin of saliva when referring to flux-induced changes of saliva ion composition. Similar to literature data, saliva sodium concentration correlated with flow rate and increased significantly during chewing; although in our study, the oral cavity sodium concentrations (3.6 to 10.3 mmol/l) remained always below of those given in literature for parotid saliva (15 to 90 mmol/l) [24].

According to Humphrey and Williamson [13], the pH value of saliva can change from 5.3 to 7.8. We observed a minimal value of 6.0 (unstimulated) and a maximal value of 8.1 (stimulated). Possibly, the method to determine pH values applied by us limited the detection of more extreme values. The calculated bicarbonate concentrations increased from 7.2 to 28.5 mmol/l, which for the stimulated saliva is still lower as the value given in literature (10–60 mmol/l) [24].

As described in the introduction, saliva production in the acini and its modification in the excretory ducts is regulated to a large extend by the autonomous nervous system [7]. To unveil possible regulation mechanisms, we had a closer look on our datasets. Besides the aggregate comparison of flow rates and ion concentrations under resting and stimulated conditions leading to a qualitative assessment (increase, decrease, no changes), our data set allowed further in-depth analysis for functional conclusions regarding possible differential and individual regulation in the acini and excretory ducts. For this purpose, the individual changes in ion concentrations and flow rates from our 102 volunteers were compared quantitatively. Our analysis unveiled that the stimulation of flow rate, which is a function of the acini, does not correlate with stimulation-induced changes in ion concentrations, which would be expected in case of a sole passive behavior of ductal transport activity. Changes in ion concentrations between secondary and primary saliva are the result of altered epithelial reabsorption and secretion in the duct system, as described in the introduction. Our results show that the ion compositions of secondary saliva in case of stimulation are not just the result of passive events in the duct system due to altered flow rate with possible overstressing of epithelial transport capacity. Rather, our data indicate an independent and individual regulation of transport mechanisms (secretion of HCO_3_^−^ and K^+^, reabsorption of Na^+^) at this site to generate the appropriate composition of oral saliva despite different flow rates. Thus, the extend of ductal transport activity changes for Na^+^, K^+^, Cl^−^, and HCO_3_^−^, but not the basic fact and direction, are regulated, at least in part, independent of the extend of acinar transport stimulation. As described in the introduction, ductal transepithelial transport processes are mediated by channels and transporters that can be regulated tightly by well-known downstream signaling cascades of the autonomous nervous system whereby all prerequisites for the individual modulation are given.

In summary, when saliva production, i.e., flow rate, increases due to stimulation the qualitative requirements for salivary ion composition (especially pH and osmolality) are met by the ability to uncouple the extend of changes in flow rate from the extend of changes in ductal ion transport. This mechanism allows to change oral delivery of proteins from the acini without possibly adverse alterations of ion composition, osmolality, or pH value.

Our analysis further revealed that the regulation of sodium and bicarbonate (or proton) transport (i.e., the changes in concentration, not the concentrations per se) is significantly coupled, whereas the regulation of potassium transport is not linked to sodium transport changes. Furthermore, the correlation of chewing-induced changes in salivary chloride concentrations, although there was no effect of stimulation on absolute chloride concentrations, indicates that changes in ductal chloride transport contribute to changes in sodium bicarbonate transport. The data obtained under real-world conditions in our study concur very well with the actual model of saliva formation derived from many experimental studies [7, 15, 18, 24]. Of course, we cannot exclude the contribution of changes in the relative contribution of different salivary glands to the final oral saliva as an additional reason for the dissociation of changes in flow rate and composition.

To identify possible reasons for the differences between our findings with those from literature, we analyzed original articles from which data were derived in more detail. We traced back the data on flow-dependent ion composition in humans to an article published in 1954 from Thaysen et al. [24]. In this study, the data presented were obtained from “three young women with uncomplicated essential hypertension” and “the flow of saliva was stimulated by subcutaneous injection of beta-methyl-acetylcholine hydrochloride.” Furthermore, parotid saliva and not oral cavity saliva, as in our study, was collected. Furthermore, the number of participants (three, and not healthy ones) was very low in comparison to our study. Another study by Bardow et al. [3] with “five healthy young males” reported for parotid saliva similar results to the ones shown by Thaysen et al. But they additionally observed that the oral cavity saliva showed quite different ion concentrations compared to parotid saliva. In that study, the potassium concentration of oral cavity saliva remained almost unaffected by flow rate at about 20 mmol/l. This is somewhat higher than the concentrations we obtained. Also, sodium concentration increased from close to zero to about 15 mmol/l, which is much less than the concentrations determined in parotid saliva (from close to zero to 80 mmol/l when stimulated). Thus, there is a clear difference in composition between the saliva released from individual glands and oral cavity saliva, as the salivary glands produce saliva of different ion compositions [8, 10, 13] that combine to oral cavity saliva. However, it is this mixture, which is relevant for saliva function.

In summary, we present data for oral cavity saliva composition based on a much larger cohort of subjects than previous studies. Our data show (i) that flow-dependence of ion concentration is heterogeneous and exists for sodium and bicarbonate, but not for potassium and chloride. Furthermore, (ii) osmolality is flow-independent; (iii) regulation of sodium, bicarbonate, and chloride transport is coupled; (iv) regulation of flow rate (Δflow rate) and ion concentrations (Δion concentration) are independent from each other; and (v) spatially separated between acini for flow rate and the duct system for ions. In addition, the differences between our results and the former ones show that it is necessary to reevaluate supposed solidified knowledge in less intensively investigated areas, especially if the original data were obtained a long time ago and are at risk of being lost or overseen.

## Data Availability

The original data are available from the authors on request
